# Acknowledgements upon Conclusion of the Pathogens Special Issue “Epidemiology, Surveillance and Control of Infectious Diseases”

**DOI:** 10.3390/pathogens12020280

**Published:** 2023-02-08

**Authors:** Cord Heuer, Magdalena Dunowska

**Affiliations:** 1 OIE Collaborating Centre for Veterinary Epidemiology and Public Health, Palmerston North P.O. Box 11222, New Zealand; 2 School of Veterinary Sciences, Massey University, Palmerston North 4441, New Zealand

One Health is a popular headline for an all-inclusive concept of our scientific work. It is not a new concept, but one along that we have been researching for many decades, if not centuries. Nevertheless, the term has become a keyword used worldwide for an inclusive approach to investigating issues such as pathogens in our various environments and host species. It was with this intention that *Pathogens* initiated this Special Issue. Epidemiology is a field that combines specialist knowledge of many disciplines, considered a facilitator of communication and various diverse skills.

We are very pleased with to present ten original publications covering the molecular properties, distribution and surveillance of pathogens and vectors. A few examples:

A sampling methodology for the surveillance of mosquitos and arboviruses in Florida was more efficient when stratified by a vertical gradient;

Genotyping parvoviruses of dogs and cats in Sri Lanka allowed identification of cats as possible source of the virus for dogs;

A hitherto unknown zoonotic risk emerged from a study of illegally traded companion animals in Italy and Austria;

A study in Barbados resulted in the identification of the seasonal and occupational distribution of human orthohantavirus infections among febrile patients;

We now know that the majority of genotypes of equine herpes virus 5 from Poland clustered within two main groups and similar viruses circulated in geographically distinct locations without any apparent age predisposition;

An investigation of re-emerging rabies virus in Southeast Asia demonstrated a need for collecting data in remote locations for detecting drivers of emergence.

In addition, three reviews detail the merits and limitations of diagnostic assays for describing the diversity of *Leptospira* spp., about virulence factors of the avian-pathogenic *Escherichia coli* (APEC) to inform antibiotic therapy, vaccine development and virulence inhibitors, and about the economic loss of the zoonotic Ross River virus (RRV) transmitted from horses to humans. A more confined investigation described an outbreak of canine distemper in a rescue centre.

This Special Issue intended to provide ‘insights into the ecological behaviour of pathogens and their impact on human and animal populations and environments’. We believe the ten publications have significantly contributed to achieving this goal. We would like to thank our colleagues and authors who collaborated and crafted articles describing their scientific thinking and outcomes.

